# Effect of peri‐operative pharmacological interventions on postoperative delirium in patients having cardiac surgery: a systematic review and Bayesian network meta‐analysis

**DOI:** 10.1111/anae.16757

**Published:** 2025-09-01

**Authors:** Ivo Queiroz, Lucas M. Barbosa, Mariano Gallo Ruelas, Beatriz Araújo, Maria L. R. Defante, Arthur H. Tavares, Cynthia Florencio de Mesquita, Tulio Pimentel, Beatriz Ximenes Mendes, Iuri Ferreira Felix, André Rivera, Rafael Oliva Morgado Ferreira, Helen M. de Oliveira, Bruno B. Righetto, Nathan J. Smischney, Guangyu Tong, Daqing Ma

**Affiliations:** ^1^ Department of Medicine APAMI Hospital Vitória de Santo Antão Brazil; ^2^ Department of Medicine Federal University of Minas Gerais Belo Horizonte Brazil; ^3^ Department of Nutrition Sciences Instituto de Investigación Nutricional Lima Peru; ^4^ Department of Medicine Nove de Julho University São Bernardo do Campo Brazil; ^5^ Department of Medicine Redentor University Center Rio de Janeiro Brazil; ^6^ Department of Medicine University of Pernambuco Recife Brazil; ^7^ Department of Medicine Federal University of Pernambuco Recife Brazil; ^8^ Department of Medicine Unichristus Fortaleza Brazil; ^9^ Department of Medicine Mayo Clinic Rochester MN USA; ^10^ Department of Medicine Federal University of Santa Catarina Florianópolis Brazil; ^11^ Department of Medicine Federal University of Mato Grosso Sinop Brazil; ^12^ Department of Medicine University Positivo Valinhos Brazil; ^13^ Department of Medicine Mayo Clinic Rochester MN USA; ^14^ Department of Medicine Yale School of Medicine New Haven CT USA; ^15^ Department of Statistics Yale School of Public Health New Haven CT USA; ^16^ Department of Anesthesiology Zhejiang University School of Medicine Children's Hospital Hangzhou China; ^17^ Department of Anaesthetics Imperial College London Chelsea and Westminster Hospital London UK; ^18^ Department of Anesthesiology The First Affiliated Hospital of Ningbo University Ningbo China

**Keywords:** dexmedetomidine, melatonin, postoperative cognitive dysfunction, postoperative delirium, systematic review cardiac surgery

## Abstract

**Introduction:**

Postoperative delirium is a common complication following cardiac surgery. Despite its known impact on patient outcomes, effective preventative strategies remain elusive. We aimed to perform a comprehensive Bayesian network meta‐analysis of randomised controlled trials assessing the effect of pharmacological interventions on the incidence of postoperative delirium.

**Methods:**

Databases were searched from inception to September 2024. Our search was updated in January 2025. Eligible studies included randomised controlled trials reporting the incidence of postoperative delirium in patients having cardiac surgery treated with pharmacological interventions. Bayesian models were used to estimate risk ratios (RR) and mean differences with 95%CrI through Markov chain Monte Carlo. Interventions were ranked using the surface under the cumulative ranking curve. Sensitivity analyses and grading of recommendations, assessment, development and evaluation assessment were conducted to evaluate the robustness and certainty of evidence.

**Results:**

Seventy‐nine randomised controlled trials comprising 24,827 patients were included, with 29 pharmacological interventions compared. Dexmedetomidine combined with melatonin was the most effective intervention, reducing the incidence of postoperative delirium compared with placebo (risk ratio 0.31, 95%CrI 0.13–0.69; low certainty). Dexmedetomidine with melatonin also significantly decreased intensive care unit stay (2.4 days, 95%CrI ‐3.50–1.10) and hospital stay (1.32 days, 95%CrI ‐2.09 to ‐0.57). Other interventions, including ketamine and risperidone, showed potential efficacy but with low or very low certainty of evidence.

**Discussion:**

We identified dexmedetomidine with melatonin as the most effective pharmacological strategy for preventing postoperative delirium following cardiac surgery. Whilst these findings highlight potential benefits, the low certainty of evidence underscores the need for more high‐quality primary evidence.

## Plain Language Summary

After heart surgery, some patients get confused or don't think clearly. This is called postoperative delirium, and it's a common problem. Even though doctors know it's bad for recovery, there aren't many proven ways to stop it. In this study, we looked at many other studies to see which medicines might help prevent this problem. We searched through medical research papers up to September 2024 and updated our search in January 2025. We only included studies where patients had heart surgery and were given medicine to help prevent delirium. We used special mathematical testing models to compare the results of different medicines and see which worked best. We found 79 good studies with a total of 24,827 patients. These studies tested 29 different medicines. The best combination was dexmedetomidine with melatonin. It helped lower the chances of confusion after surgery. Patients who took this combination also stayed for a shorter time in the intensive care unit and hospital. Some other medicines, like ketamine and risperidone, may help too, but we're not very sure about their results. Dexmedetomidine with melatonin seems to be the best medicine mix for stopping confusion after heart surgery. But, because we're not 100% sure about all the results, we still need more strong studies to be certain.

## Introduction

Postoperative delirium is a common complication following cardiac surgery and is associated with increased mortality and prolonged hospital stay [1]. Despite its significant impact on patient outcomes, effective strategies for its prevention remain elusive. Pharmacological interventions targeting underlying mechanisms such as inflammation, neurotransmitter imbalances and metabolic dysregulation have shown some promise [2], but a comprehensive understanding of their comparative efficacy is lacking.

Several measures to reduce the incidence of postoperative delirium have been proposed. The European Society of Anesthesiology and Intensive Care Medicine updated its guidelines recently [3]. Dexmedetomidine was the only pharmacological intervention recommended for the treatment of postoperative delirium in patients having cardiac surgery. However, the strength of this recommendation was weak, based on the very low‐quality evidence. The mechanism of the effect of α 2‐adrenoreceptor agonists on delirium is likely related to their anxiolytic and sedative properties as well as a decrease in sympathetic tone. These guidelines also suggested haloperidol for the treatment of postoperative delirium in adult patients, albeit based on very low quality of evidence. The mechanism of the effect of antipsychotics on delirium likely relates to the management of psychotic symptoms [3]. Recently, the American Society of Anesthesiologists released a practice advisory on strategies to reduce the risk of postoperative delirium in older patients (aged ≥ 65 y) having inpatient surgery [4]. Among various recommendations such as minimising medications with potential central nervous system effects and expanded pre‐operative evaluation, dexmedetomidine was conditionally recommended to lower the risk of postoperative delirium for older adult inpatients having surgery. This was based on moderate evidence and no other pharmacological interventions were recommended [4].

Although these recommendations do not endorse ketamine for either prevention or treatment of postoperative delirium in adults, a recent randomised controlled trial showed that ketofol (combination of ketamine and propofol) was as effective as dexmedetomidine in reducing postoperative delirium in patients aged ≥ 60 y undergoing urgent abdominal exploration for intestinal obstruction [5]. Ketofol has also been shown to be associated with lower postoperative delirium rates in children following surgery [6, 7]. The ketamine component, acting via the N‐methyl‐D‐aspartate (NMDA) receptor, is believed to be the mechanism behind the reduction in postoperative delirium. However, not all studies evaluating ketamine and postoperative delirium have shown promising results [8, 9]. A possible explanation may relate to ketamine dosing and the plausible effect of propofol acting as a neuroprotective drug (anti‐inflammation, anti‐oxidative, anti‐apoptosis, etc.) [10].

To our knowledge, this is the first comprehensive network meta‐analysis assessing the incidence of postoperative delirium in patients undergoing cardiac surgery. Our study aimed to synthesise evidence on pharmacological interventions for postoperative delirium prevention in patients having cardiac surgery. The network meta‐analysis not only consolidates existing knowledge but also generates clinically meaningful insights into the efficacy and safety of these interventions. We provide clinicians and researchers with a comprehensive evaluation of available pharmacological options for postoperative delirium prevention following cardiac surgery, highlighting promising strategies and identifying future areas of research.

## Methods

We searched PubMed, Embase and Cochrane databases, with the first search conducted in September 2024 and a final update in January 2025. Our study was conducted according to PRISMA guidelines [11, 12, 13]. Studies from database inception until January 2025 were searched (online Supporting Information Table S1). Three authors reviewed the retrieved records independently. Decisions regarding the inclusion of full‐text articles were made through consensus. Subsequently, three authors reviewed the full texts, with detailed discussions on inclusion and exclusion criteria. Reference lists of eligible studies and systematic reviews were also screened to identify additional articles. The search encompassed prospective trials to ensure comprehensive coverage.

Studies were eligible if they were: randomised controlled trials; reported the incidence of delirium in a postoperative setting; used a pharmacological drug in any cardiac surgery setting; and were published in a peer‐reviewed journal. The primary outcome was the incidence of postoperative delirium, assessed in studies typically until ICU discharge or within 7 days postoperatively. A minority of trials reported shorter follow‐ups of 3–5 postoperative days. Secondary outcomes included: intra‐operative mortality; 30‐day mortality; incidence of postoperative acute kidney injury; ICU duration of stay; duration of hospital stay; and time to tracheal extubation.

Three authors extracted data from each eligible study on each outcome independently, such as the specifics of interventions and comparators, the incidence of outcomes in each group and the mean difference for continuous outcomes. Furthermore, three authors collected information about the studies (e.g. bibliographic information, country of origin and funding source) and patient characteristics (e.g. sample size, age, sex, cardiac surgery type, baseline comorbidities and surgery duration). Data were extracted for all intervention characteristics clinically meaningful to patients, such as how and when drugs were administered.

We performed a Bayesian network meta‐analysis [14] for continuous and binary outcomes using the gemtc package in R (version 4.4.1) [15, 16]. The model was estimated using the Markov chain Monte Carlo method [17]. Convergence was checked using trace plots and Gelman‐Rubin diagnostics [18]. The model included non‐informative priors for the mean and variance parameters with four parallel chains each with 100,000 iterations. Heterogeneity between the direct and indirect comparisons was assessed using the node‐split plots. We ranked the interventions by using the surface under the cumulative ranking curve [19]. Studies with broad credible intervals due to rare events (upper boundary > 100, lower boundary < 0.001) were not included in the main Bayesian analysis. Studies reporting ICU and duration of hospital stay in hours were standardised to days for consistency before pooling. For continuous outcomes expressed as median (IQR [range]), these were converted to mean (SD) using the Wan and Luo method [20], ensuring comparability across the dataset.

A frequentist network meta‐analysis [14] was performed to assess the comparative effect of interventions with rare frequency of events, and p‐scores were compared with surface under the cumulative ranking curve values to compare the ranking of the interventions under different statistical approaches. Two authors assessed the risk of bias in the included trials using the Cochrane tool [21]. Disagreements were resolved through consensus. The exploration of publication bias was performed only for the main outcome with a funnel plot and Egger's test [22].

We applied the GRADE framework to evaluate the certainty of evidence across direct, indirect and network estimates for the main outcomes. This approach begins with direct evidence from randomised trials classified as high certainty, which can be downgraded to moderate, low or very low based on factors such as risk of bias, indirectness, imprecision, inconsistency or small‐study effects. For indirect estimates, the initial certainty level corresponds to the lowest GRADE rating among the direct comparisons that predominantly influenced the key loop in the network, with further downgrading if intransitivity was observed [23].

To evaluate transitivity, we considered two critical aspects: whether the trials involved could be randomised collectively; and whether effect modifiers were distributed evenly across treatment comparisons in the network. To address the first criterion, we ensured the patient populations in the trials were comparable and confirmed with clinical experts that all interventions in the network could have been applied to any eligible patients. For the second criterion, we analysed graphical representations of potential effect modifiers, such as age, sex and risk of bias, to confirm their similarity across comparisons. Given the uniform randomised trial design and specific population of patients having cardiac surgery, transitivity was not expected to pose a concern.

For network estimates, we determined the starting certainty level based on the relative contribution of direct or indirect evidence. If one source contributed > 50%, its certainty level was used as the foundation. When both contributed equally, we used the higher certainty of the two. Imprecision was assessed at the network level and judged by whether the 95% credible interval (95%CrI) encompassed decision thresholds. The guideline panel predefined these thresholds as half the minimally significant difference for continuous outcomes or the null value (RR = 1) for postoperative delirium, mortality and acute kidney injury. If the 95%CrI did not include these thresholds, we did not downgrade for imprecision, except in cases where the comparison was based on < 400 patients for continuous outcomes or 300 events for binary outcomes. However, due to the inherent difference in dosages and administration protocols for the evaluated drugs, imprecision or indirectness was expected to be downgraded for most comparisons.

When incoherence arose in network estimates, we prioritised using the three comparisons to estimate the certainty. We avoided double downgrading for intransitivity and incoherence; the same was valid for imprecision and indirectness when associated with a similar factor. For consistency in presenting our findings, we adhered to GRADE guidelines and followed the grading method reported by Izcovich et al. [24].

## Results

**Figure 1:**
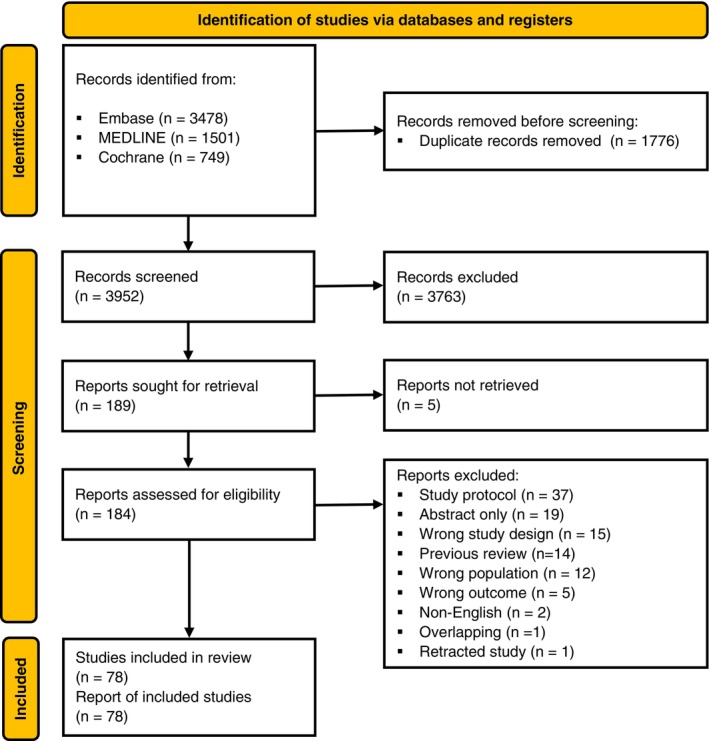
Study flow diagram.

We found 5728 published articles, of which 1776 were duplicates and 3763 were excluded due to not being related or being unable to be used in our analysis. This left 189 articles which were read in full, with 78 (41%) [25, 26, 27, 28, 29, 30, 31, 32, 33, 34, 35, 36, 37, 38, 39, 40, 41, 42, 43, 44, 45, 46, 47, 48, 49, 50, 51, 52, 53, 54, 55, 56, 57, 58, 59, 60, 61, 62, 63, 64, 65, 66, 67, 68, 69, 70, 71, 72, 73, 74, 75, 76, 77, 78, 79, 80, 81, 82, 83, 84, 85, 86, 87, 88, 89, 90, 91, 92, 93, 94, 95, 96, 97, 98, 99, 100, 101] randomised controlled trials included (Fig. 1, online Supporting Information Table S2 and Appendix S1). All the included trials reported the incidence of postoperative delirium, usually until ICU discharge or 7 days postoperatively. A small number of trials reported a shorter follow‐up of 3–5 days. The intervention was administered in the pre‐operative and intra‐operative period in 45 trials; postoperative period in 20 trials; and intra‐operative period in 14 trials.

Of the 79 trials, 37 (48%) showed risk of bias in at least one domain; 41 (52%) properly generated their randomisation sequence; 69 (87%) implemented adequate allocation concealment; 76 (96%) had no issues with missing outcomes; and 63 (80%) had no biases associated with outcome assessment. Seventy‐four (94%) trials reported < 20% missing outcome data or no evidence of selective reporting. Overall, we assessed the risk of bias as high in 9 (11%) studies and low or probably low in 41 (52%) (online Supporting Information Table S3).

**Figure 2:**
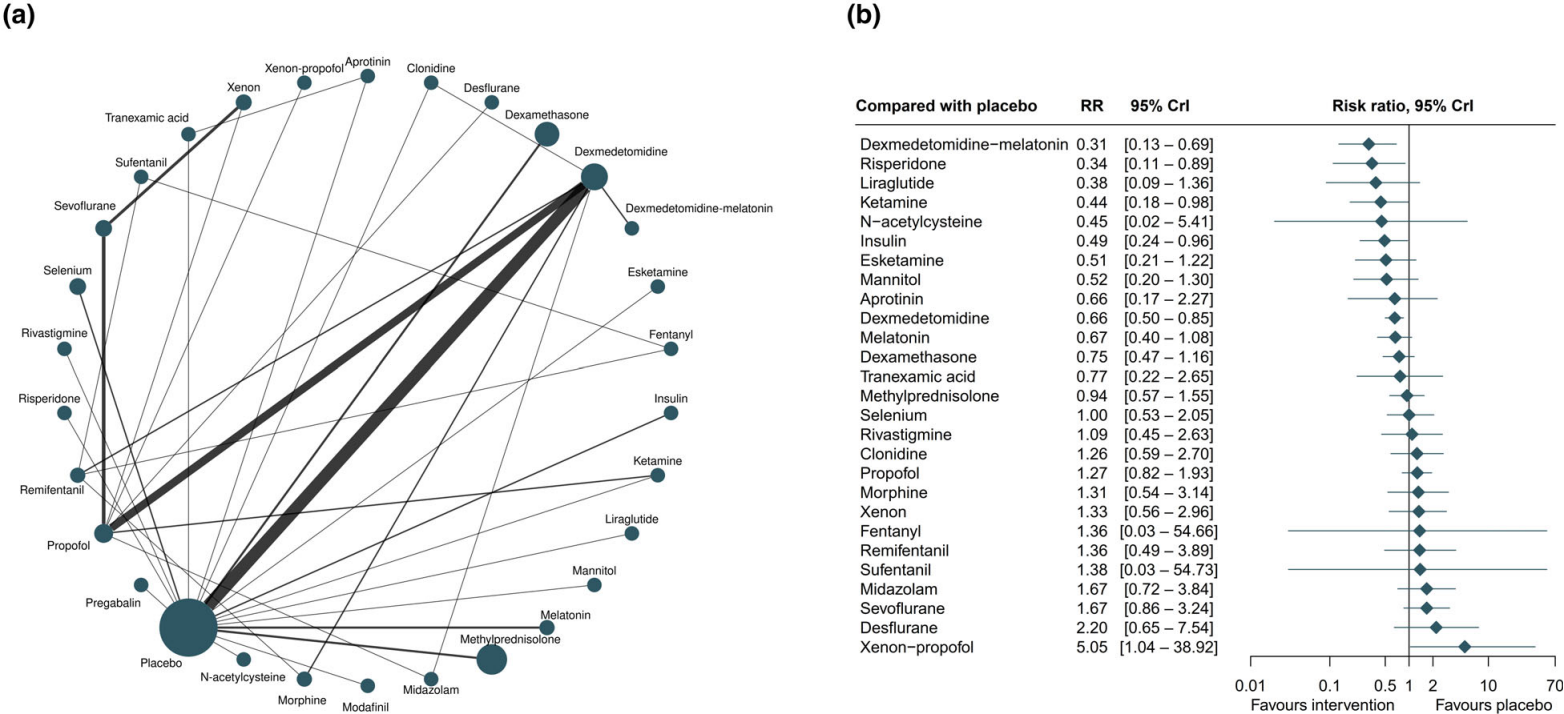
Network plot for postoperative delirium (a) and interventions for postoperative delirium compared with placebo (b). RR, risk ratio.

In total, 79 randomised controlled trials including 24,827 patients and evaluating 29 interventions reported their effects on delirium (online Supporting Information Appendix S2). The network plot for the incidence of postoperative delirium revealed that dexmedetomidine was the drug featuring in the highest number of randomised controlled trials (Fig. 2A). The direct comparison of dexmedetomidine with melatonin vs. dexmedetomidine and indirect comparisons with other interventions showed that dexmedetomidine with melatonin was the most likely intervention to reduce the incidence of postoperative delirium as compared with placebo, although with low certainty (Table 1).

**Table 1:** Summary of findings and grading of recommendations assessment, development and evaluation.

Interventions compared	Relative effect (95%CrI)	Studies (patients)*	Evidence used**	Certainty of evidence***	SUCRA (%)
Dexmedetomidine with melatonin vs. dexmedetomidine	0.47 (0.21–1.01)	2 (3199)	Direct	Moderate	‐
Dexmedetomidine vs. placebo	0.66 (0.50–0.85)	18 (13,693)	NMA	Low	9 (0.65%)
Dexmedetomidine with melatonin vs. placebo	0.31 (0.13–0.69)	2 (10,515)	Indirect	Low	1 (0.88%)
Risperidone vs. placebo	0.34 (011–0.89)	1 (10,489)	Direct	Low	2 (0.85%)
Insulin vs. placebo	0.49 (0.24–0.96)	2 (10,540)	Direct	Low	5 (0.76%)
Dexmedetomidine vs. risperidone	1.96 (0.70–5.79)	18 (3173)	Indirect	Low	‐
Ketamine vs. dexmedetomidine	0.67 (0.28–1.45)	18 (3196)	Indirect	Very low	‐
Ketamine vs. placebo	0.44 (0.18–0.98)	1 (10,512)	NMA	Very low	3 (0.79%)
Dexmedetomidine with melatonin vs. ketamine	0.71 (0.23–2.25)	3 (175)	Indirect	Very low	‐

NMA, network meta‐analysis; SUCRA, surface under the cumulative ranking curve.

* Data from intervention plus comparison.

** Type of comparison.

*** Final rating of certainty.

Compared with placebo, low certainty evidence supported the use of five interventions to prevent postoperative delirium: dexmedetomidine with melatonin (RR 0.31, 95%CrI 0.13–0.69, low certainty of evidence); ketamine (RR 0.44, 95%CrI 0.18–0.98, very low certainty of evidence); risperidone (RR 0.34, 95%CrI 0.11–0.89, low certainty of evidence); insulin (RR 0.49, 95%CrI 0.24–0.96, low certainty of evidence); and dexmedetomidine (RR 0.66, 95%CrI 0.50–0.85, low certainty of evidence) (Fig. 2b).

**Figure 3:**
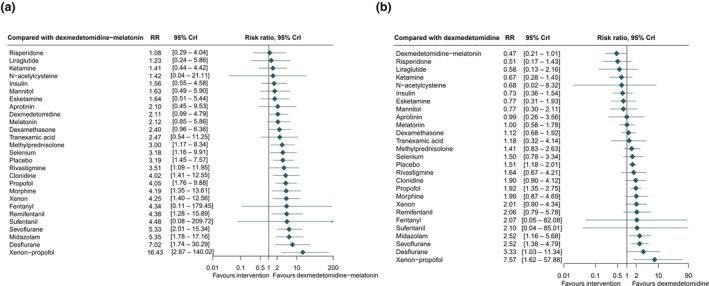
Interventions for postoperative delirium compared with dexmedetomidine‐melatonin (a) and dexmedetomidine (b). RR, risk ratio.

When dexmedetomidine with melatonin was compared indirectly with other interventions, it indicated superiority (Fig. 3a), although the wide 95%CrI reflected reliance on indirect comparisons. Other interventions showed potential for reducing postoperative delirium compared with placebo but crossed the CrI. These interventions included: liraglutide (RR 0.38, 95%CrI 0.09–1.36); esketamine (RR 0.51, 95%CrI 0.51–1.22); mannitol (RR 0.52, 95%CrI 0.20–1.30); and aprotinin (RR 0.66, 95%CrI 0.17–2.27) (Fig. 2b). Ketamine and risperidone did not show superiority over most interventions in their respective comparison plots (online Supporting Information Figure S1).

Eight interventions contributed to the assessment of mortality against placebo (online Supporting Information Figures S2A and S2B) with 6771 patients and 10 randomised controlled trials. No intervention was associated with an improvement or increased mortality risk. Most interventions showed high 95%Crl intervals, but this was expected as most studies focused on delirium prevention.

There were 19 randomised controlled trials including 7307 patients available for the analysis of the incidence of acute kidney injury (online Supporting Information Figure S3A). There were nine interventions for this outcome that contributed to comparisons against placebo. No comparator was associated with an increased acute kidney injury incidence vs. placebo (online Supporting Information Figure S3B).

Forty‐two randomised controlled trials contributed to the analysis of ICU duration of stay, with a total of 6027 patients and 21 interventions (online Supporting Information Figure S3C). Only dexmedetomidine with melatonin had a significant result (mean difference ‐2.4 days, 95%CrI ‐3.50 to ‐1.10) days, showing that it decreased ICU stay for approximately 2 days compared with placebo. Other interventions showed no associations with this outcome (online Supporting Information Figure S3D). Forty‐two randomised controlled trials comprising 5910 patients and 21 interventions were compared in the assessment of duration of hospital stay (online Supporting Information Figure S4A). Dexmedetomidine with melatonin was the only intervention to show a significant effect, reducing hospital stay by approximately 1 day with a mean difference of ‐1.32 (95%CrI ‐2.09 to ‐0.57) days (online Supporting Information Figure S4B). Fourteen randomised controlled trials with a total of 2569 patients were analysed for time to tracheal extubation (online Supporting Information Figure S4C). Notably, none of the comparators showed an association with a decreased risk of time to tracheal extubation (online Supporting Information Figure S4D).

**Figure 4:**
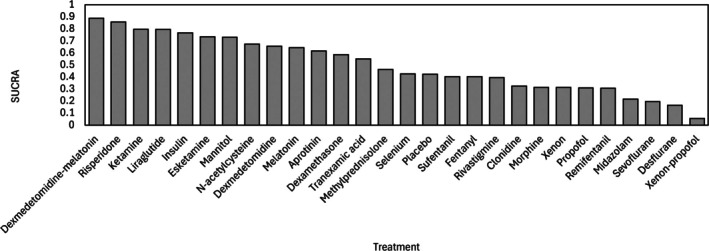
Surface under the cumulative ranking curve (SUCRA) for postoperative delirium.

Dexmedetomidine combined with melatonin had the highest surface under the cumulative ranking curve value (0.887), highlighting it as the most effective intervention. Risperidone (0.856) and ketamine (0.796) also stand out as promising options. In contrast, interventions such as placebo (0.423), midazolam (0.216) and xenon with propofol (0.056) had lower values, suggesting comparatively lower efficacy. This ranking supports therapeutic decision‐making by prioritising interventions with superior performance in preventing postoperative delirium (Fig. 4a). The sensitivity analysis confirmed the robustness of our findings, with consistent ranking estimates across various statistical methods reinforcing the reliability of our findings.

The funnel plot assessing publication bias showed most of the effect sizes fell inside the funnel triangle (online Supporting Information Figure S5). However, slight asymmetry in the lower‐right quadrant indicates potential small‐study effects, as supported by Egger's test (p = 0.013).

**Figure 5:**
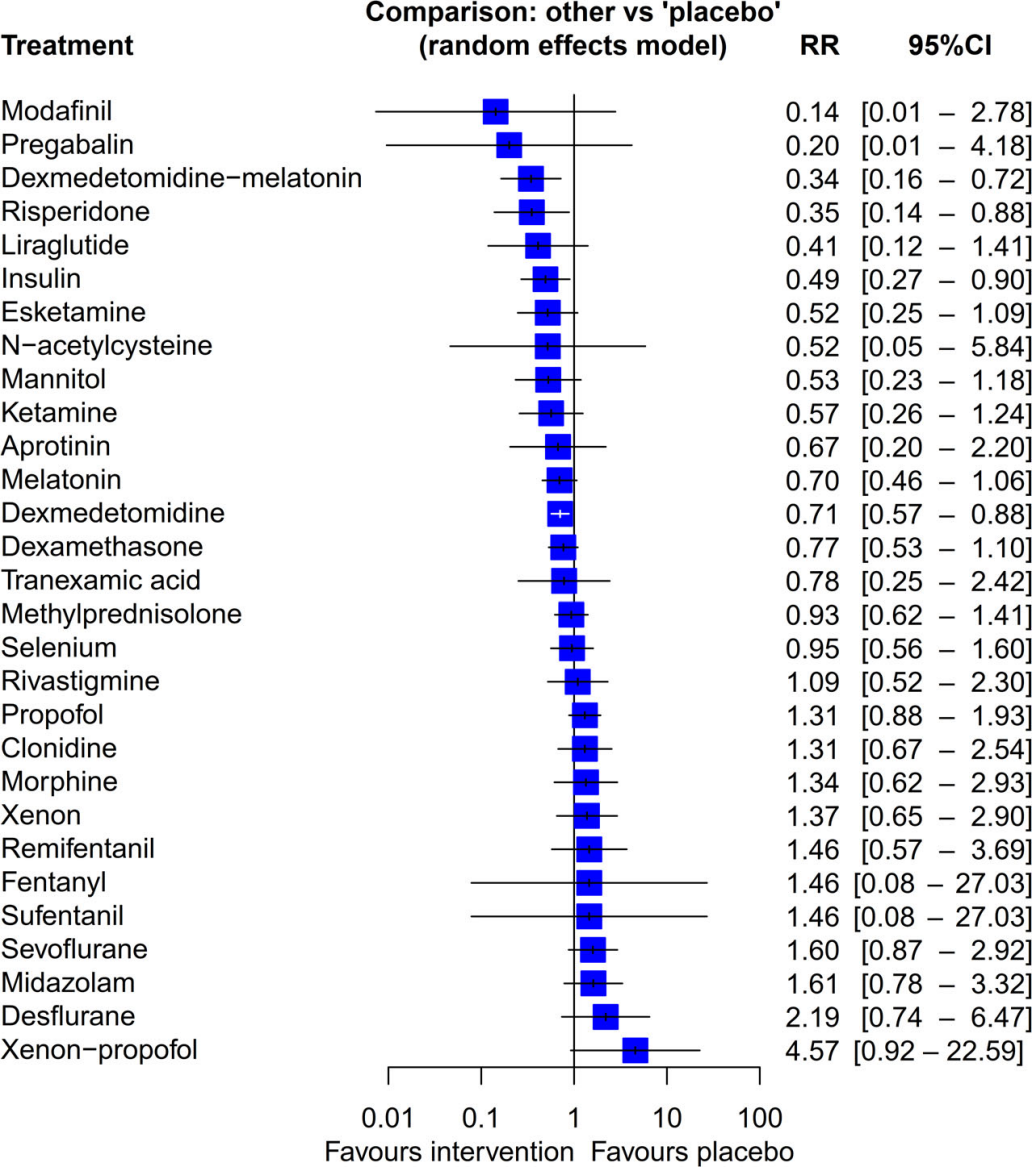
Frequentist network meta‐analysis of comparisons against placebo. RR, risk ratio.

The frequentist network meta‐analysis P‐scores ranked interventions by efficacy (Table 2). Dexmedetomidine combined with melatonin achieved the highest P‐score (0.8432), indicating superior effectiveness. Modafinil (0.8619) and risperidone (0.8614) also ranked highly but showed no statistically significant differences compared with placebo in the frequentist pairwise comparison (Fig. 5). Conversely, xenon‐propofol (0.0497) and desflurane (0.1207) had the lowest P‐scores, suggesting limited efficacy.

**Table 2:** Frequentist sensitivity analysis. P‐score for postoperative delirium.

	P‐score (common)	P‐score (random)
Dexmedetomidine‐melatonin	0.8432	0.8534
Modafinil	0.8619	0.8505
Risperidone	0.8614	0.8350
Pregabalin	0.8081	0.7952
Liraglutide	0.7951	0.7680
Insulin	0.7836	0.7552
Esketamine	0.7604	0.7250
Mannitol	0.7506	0.7158
Ketamine	0.6393	0.6919
N‐acetylcysteine	0.6468	0.6284
Dexmedetomidine	0.5757	0.6151
Melatonin	0.6420	0.6141
Aprotinin	0.6274	0.5980
Dexamethasone	0.5854	0.5664
Tranexamic acid	0.5640	0.5356
Methylprednisolone	0.4482	0.4530
Selenium	0.5064	0.4455
Placebo	0.4265	0.4106
Rivastigmine	0.3995	0.3779
Fentanyl	0.3427	0.3699
Sufentanil	0.3427	0.3699
Clonidine	0.2708	0.2921
Morphine	0.2584	0.2877
Propofol	0.2577	0.2829
Xenon	0.2193	0.2797
Remifentanil	0.2167	0.2652
Midazolam	0.1949	0.2093
Sevoflurane	0.2011	0.2001
Desflurane	0.1207	0.1477
Xenon‐propofol	0.0497	0.0611
Desflurane	0.1207	0.1477
Xenon‐propofol	0.0497	0.0611

Modafinil (RR 0.14, 95%CI 0.01–2.78) and pregabalin (RR 0.20, 95%CI 0.01–4.18) reduced postoperative delirium risk, but no statistical sigificance was found. Dexmedetomidine plus melatonin (RR 0.34, 95%CI 0.16–0.72) showed substantial efficacy. Risperidone (RR 0.35, 95%CI 0.14–0.88) and ketamine (RR 0.52, 95%CI 0.26–1.24) showed moderate effects. In contrast, xenon‐propofol (RR 4.57, 95%CI 0.92–22.59) and desflurane (RR 2.19, 95%CI 0.74–6.47) were less effective, favouring placebo (Fig. 5).

Comparing these results with the surface under the cumulative ranking curve results and Bayesian forest plot, we observed consistent trends. Interventions such as dexmedetomidine with melatonin, modafinil and risperidone consistently ranked highly across both analyses, indicating their potential effectiveness in reducing postoperative delirium incidence. Conversely, treatments like xenon with propofol and desflurane were consistently ranked lower, suggesting limited efficacy. These consistent findings across different analytical methods reinforce the robustness of our conclusions regarding the relative effectiveness of these interventions. Collectively, these analyses validate the efficacy rankings of interventions for postoperative delirium. While interventions like dexmedetomidine with melatonin, modafinil and risperidone appear effective, potential biases and variability in lower‐ranked treatments underscore the need for further research.

The combination of dexmedetomidine and melatonin showed a strong effect but was rated as low certainty, primarily due to indirectness and imprecision. Dexmedetomidine alone showed a reduction in postoperative delirium, also rated as low certainty, while ketamine was classified as very low certainty due to inconsistency and imprecision (Table 1). Risperidone showed potential for reducing postoperative delirium but was rated as low certainty due to the risk of bias and imprecision. Insulin was rated as low certainty due to indirectness. The combination of dexmedetomidine with melatonin vs. dexmedetomidine had moderate certainty and showed statistically significant effects on the frequentist analysis, suggesting it may be a viable strategy, though further studies are needed. Conversely, comparisons such as dexmedetomidine with melatonin vs. ketamine, dexmedetomidine vs. risperidone and ketamine vs. dexmedetomidine were rated as low or very low certainty due to significant inconsistency and imprecision, making the findings inconclusive.

## Discussion

We found that the combination of dexmedetomidine and melatonin reduced the incidence of postoperative delirium significantly compared with other treatments. This combination was the only intervention that supported a statistically significant lower incidence of postoperative delirium compared with dexmedetomidine alone. These results were consistent across various clinical outcomes, including duration of hospital and ICU stay and time to tracheal extubation, where the dexmedetomidine with melatonin combination achieved the highest surface under the cumulative ranking curve scores, trailing only behind tranexamic acid for time to tracheal extubation. However, we found no pharmacological regimen that significantly reduced the incidence of mortality or acute kidney injury in this analysis.

The exact underlying mechanisms of postoperative delirium remain unknown. Genetic predisposition; pathogenesis of neurodegenerative disorders; vascular diseases; peri‐operative hypotension; thromboembolism; reperfusion injury; neuronal metabolism; neuroinflammation; and blood–brain barrier breakdown have all been associated with the occurrence and development of postoperative delirium [102]. Inflammatory responses induced by surgical trauma eventually reaching the brain cause various changes from molecular, cellular to brain neural‐circuits and even regional brain structure changes [103]. In addition, gut microbial dysbiosis has been reported after surgical disruption of the intestinal barrier and metabolic abnormalities, resulting in neuroinflammation and dendritic spine loss [104]. Gut microbial dysbiosis and intestinal barrier injury yield endotoxemia, systemic inflammation and brain function impairment, in particular where brain function was already vulnerable [105].

All these pathological changes trigger mitochondria dysfunction; oxidative stress mediated neuronal damage; microglia synaptic elimination; and neuronal pyroptosis, and contribute to postoperative delirium occurrence. Furthermore, due to pain, anxiety, stress and peri‐operative sleep deprivation, most patients, especially older patients, have sleep disorders before and after surgery, which induces postoperative delirium [106]. Dexmedetomidine is anti‐inflammatory, anti‐stress and neuroprotective in addition to its analgesic and sleep‐aiding effects [107]. A previous meta‐analysis also found that dexmedetomidine decreased the incidence of postoperative delirium in patients undergoing cardiac surgery [108]. This may explain the combination of dexmedetomidine and melatonin being superior to other pharmacological interventions to prevent postoperative delirium development and occurrence.

However, in our network meta‐analysis, the primary challenges in the existing literature were not necessarily the lack of randomised controlled trials but rather the variability in dosing regimens and inconsistencies in intervention timing. Indeed, most studies that used dexmedetomidine had different dosages and protocols, making indirectness a major concern. Overall, our findings were to be expected due to the high clinical heterogeneity in pharmacological interventions in the peri‐operative scenario. Despite limitations, dexmedetomidine combined with melatonin appears to be the most effective intervention based on the available data. Although the certainty of evidence is low, its consistent performance across various outcomes and mechanistic rationale supports its use in most patients. Considering individual patient characteristics and institutional expertise, dexmedetomidine with melatonin can be recommended as a preferred approach when feasible while awaiting further high‐quality evidence.

Our study is the first comprehensive Bayesian network meta‐analysis to identify improved outcomes with dexmedetomidine combined with melatonin for the prevention of postoperative delirium following cardiac surgery. In the absence of more definitive evidence, a preference for this pharmacological combination may be justified, provided it aligns with institutional protocols, clinician expertise and patient‐specific factors. Nevertheless, the current evidence does not address several relevant variables and methodological limitations adequately, which constrain the certainty of these findings. The low certainty of evidence and high variability in pharmacological protocols underscores the need for future high‐quality research. A well‐designed multicentre randomised controlled trial with a standardised protocol for drug administration, clear outcome definitions and sufficient statistical power would improve the evidence base. Such a trial should compare interventions such as dexmedetomidine‐melatonin, dexmedetomidine alone and other promising drugs to establish definitive recommendations.

## Supporting information


**Plain Language Summary**.

**Appendix S1.** Characteristics of included studies.

**Appendix S2.** League table showing the comparisons of interventions for postoperative delirium.

**Figure S1.** Interventions for postoperative delirium compared with ketamine and risperidone.
**Figure S2.** Network plot for mortality and interventions for mortality compared with placebo.
**Figure S3.** Network plot for acute kidney injury; interventions for acute kidney injury compared with placebo; network plot for intensive care unit duration of stay; and interventions for intensive care unit duration of stay compared with placebo.
**Figure S4.** Network plot for hospital duration of stay; interventions for hospital duration of stay compared with placebo; network plot for time to tracheal extubation; and interventions for time to tracheal extubation compared with placebo.
**Figure S5.** Funnel plot for postoperative delirium.

**Table S1.** Search strategy for each database.
**Table S2.** Baseline characteristics of included studies.
**Table S3.** Critical appraisal of individual studies.
