# Supplementary material for: Effect of peri‐operative pharmacological interventions on postoperative delirium in patients having cardiac surgery: a systematic review and Bayesian network meta‐analysis
Source: Anaesthesia. 2025 Sep 1;81(2):274–87. doi: 10.1111/anae.16757 (PMC12803695; doi:10.1111/anae.16757)
Supplement: Supplementary file 1 — Plain Language Summary. [file ANAE-81-274-s004.docx]

**Plain Language Summary**

After heart surgery, some patients get confused or don’t think clearly. This is called postoperative delirium, and it’s a common problem. Even though doctors know it’s bad for recovery, there aren’t many proven ways to stop it. In this study, we looked at many other studies to see which medicines might help prevent this problem. We searched through medical research papers up to September 2024 and updated our search in January 2025. We only included studies where patients had heart surgery and were given medicine to help prevent delirium. We used special mathematical testing models to compare the results of different medicines and see which worked best. We found 79 good studies with a total of 24,827 patients. These studies tested 29 different medicines. The best combination was dexmedetomidine with melatonin. It helped lower the chances of confusion after surgery. Patients who took this combination also stayed for a shorter time in the intensive care unit and hospital. Some other medicines, like ketamine and risperidone, may help too, but we’re not very sure about their results. Dexmedetomidine with melatonin seems to be the best medicine mix for stopping confusion after heart surgery. But, because we’re not 100% sure about all the results, we still need more strong studies to be certain.
