# Supplementary material for: Effect of peri‐operative pharmacological interventions on postoperative delirium in patients having cardiac surgery: a systematic review and Bayesian network meta‐analysis
Source: Anaesthesia. 2025 Sep 1;81(2):274–87. doi: 10.1111/anae.16757 (PMC12803695; doi:10.1111/anae.16757)
Supplement: Supplementary file 4 — Figure S1. Interventions for postoperative delirium compared with ketamine and risperidone. Figure S2. Network plot for mortality and interventions for mortality compared with placebo. Figure S3. Network plot for acute kidney injury; interventions for acute kidney injury compared with placebo; network plot for intensive care unit duration of stay; and interventions for intensive care unit duration of stay compared with placebo. Figure S4. Network plot for hospital duration of stay; interventions for hospital duration of stay compared with placebo; network plot for time to tracheal extubation; and interventions for time to tracheal extubation compared with placebo. Figure S5. Funnel plot for postoperative delirium. [file ANAE-81-274-s001.docx]

**Figure S1**. Interventions for post-operative delirium compared with ketamine (A) and risperidone (B).


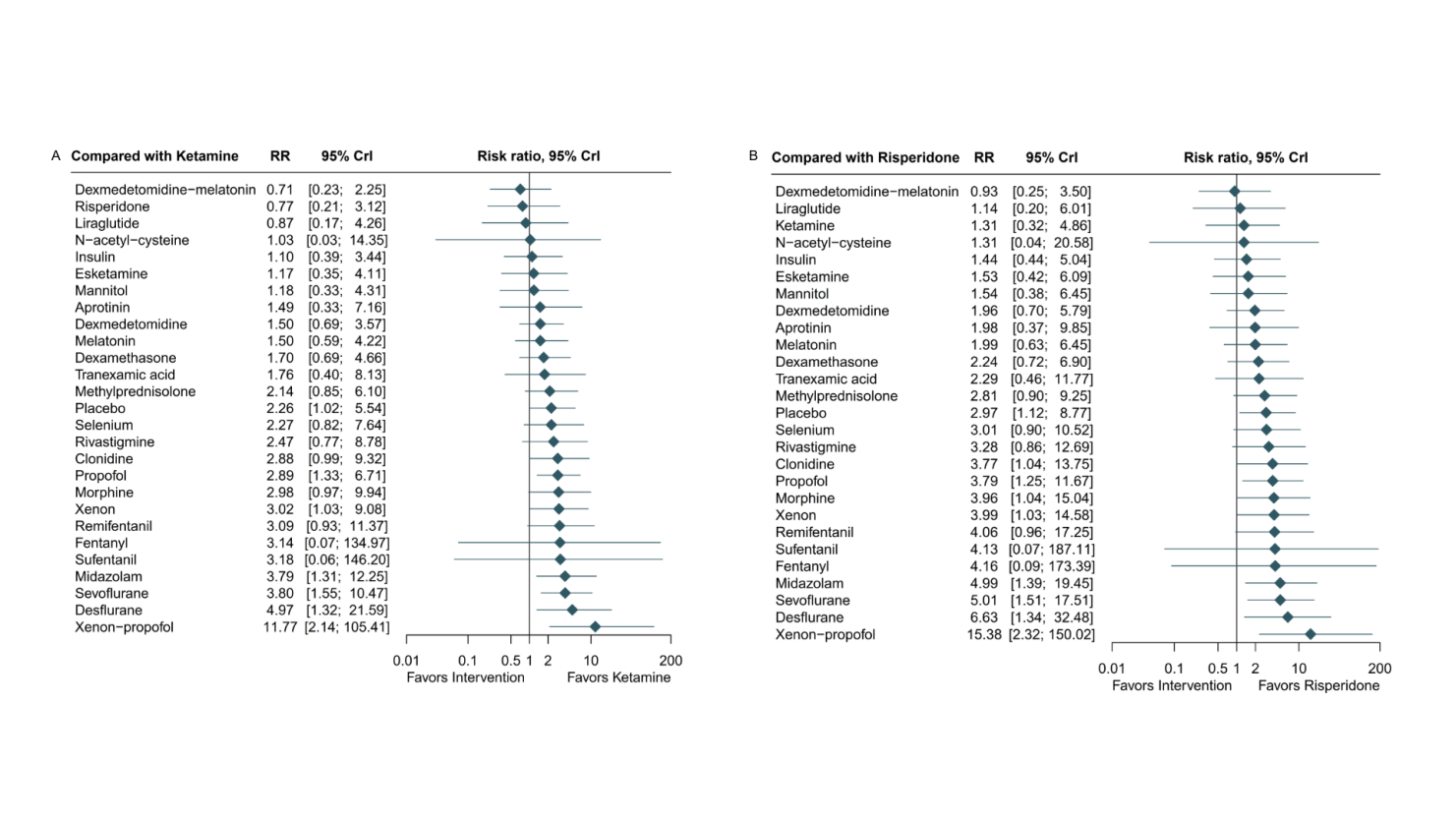


**Figure S2**. Network plot for mortality (A) and interventions for mortality compared with placebo (B).


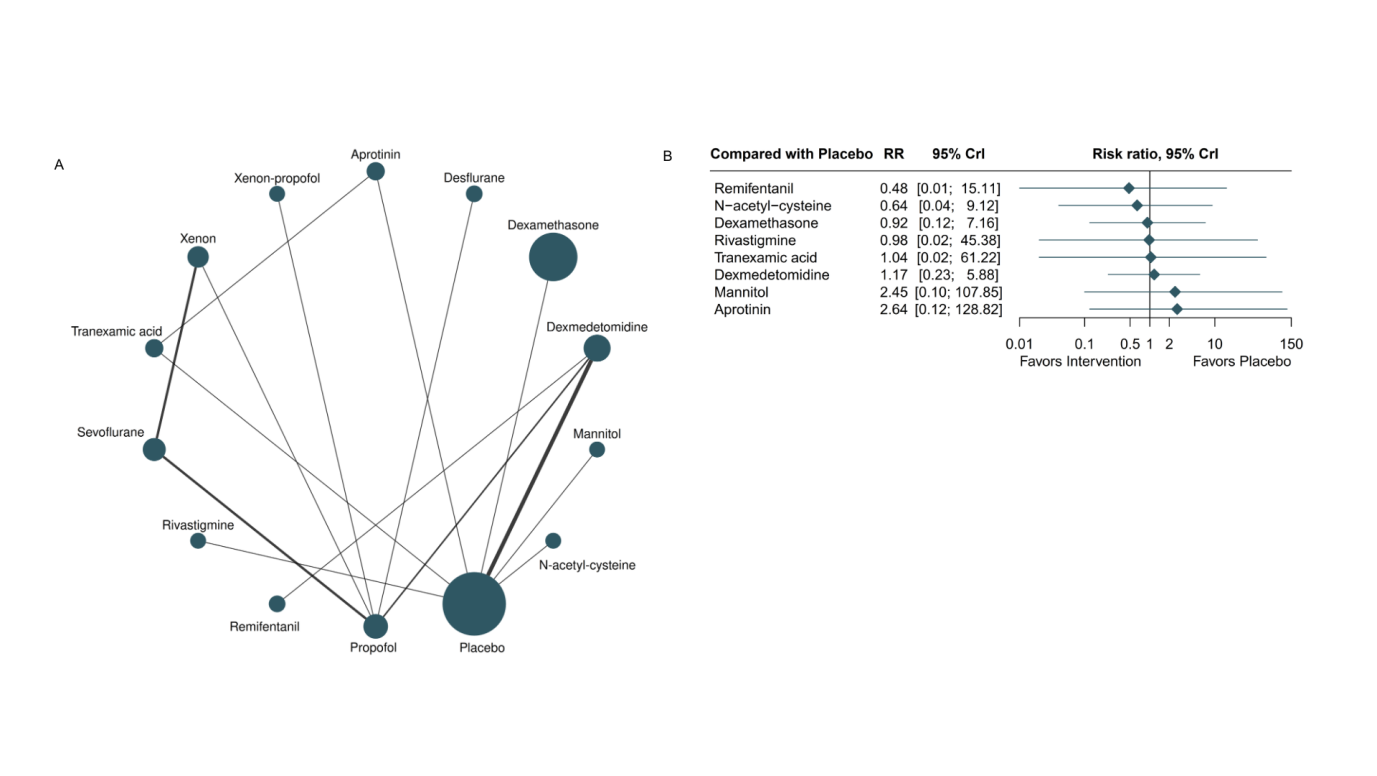


**Figure S3**. Network plot for acute kidney injury (A), interventions for acute kidney injury compared with placebo (B), network plot for intensive care unit length-of-stay (C) and interventions for intensive care unit length-of-stay compared with placebo (D).


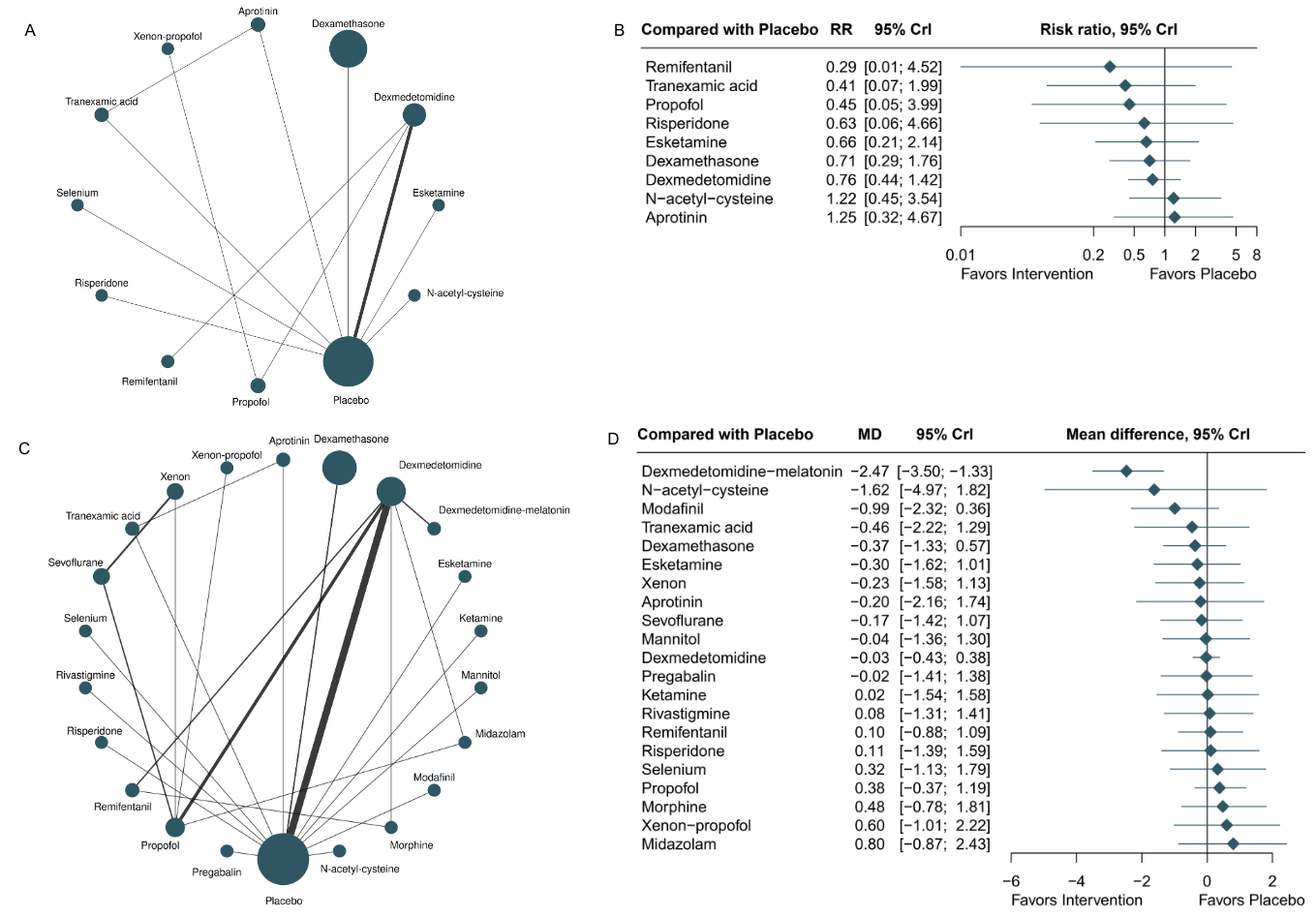


**Figure S4**. Network plot for hospital length-of-stay (A), interventions for hospital length-of-stay compared with placebo (B), network plot for time to extubation (C) and interventions for time to extubation compared with placebo (D).


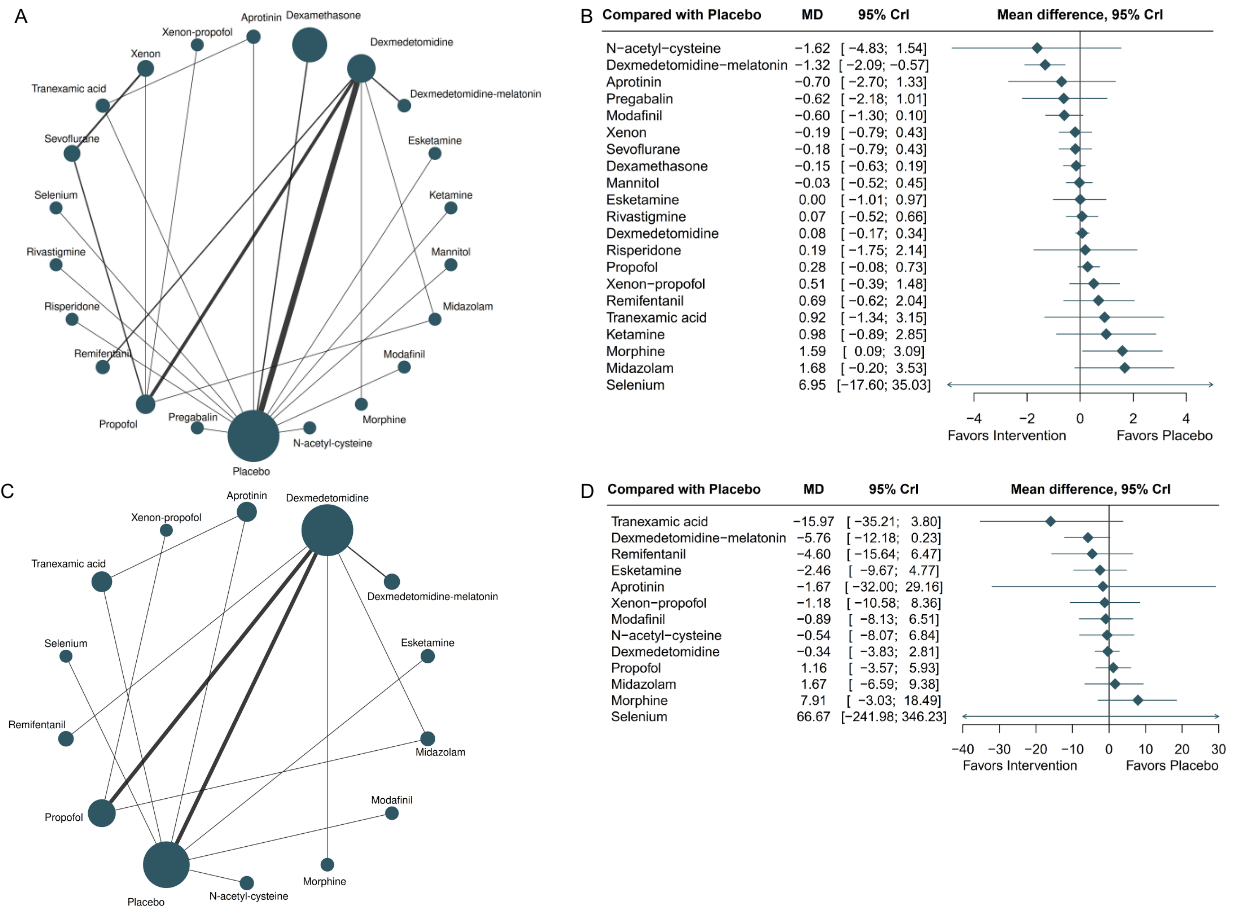


**Figure S5** Funnel plot for postoperative delirium. Aprotinin vs. placebo: blue; aprotinin vs. tranexamic acid: brown; clonidine vs. dexmedetomidine: purple; clonidine vs. placebo: green; desflurane vs. propofol: grey; dexamethasone vs. placebo: yellow; dexmedetomidine vs. dexmedetomidine−melatonin: orange; dexmedetomidine vs. midazolam: dark blue; dexmedetomidine vs. morphine: pink; dexmedetomidine vs. placebo: red; dexmedetomidine vs. propofol: black; dexmedetomidine vs. remifentanil: pink; esketamine vs. placebo: dark green; fentanyl vs. remifentanil: dark orange; fentanyl vs. sufentanil: hot pink; insulin vs. placebo: cyan; ketamine vs. placebo: cyan; ketamine vs. propofol: dark goldenrod; liraglutide vs. placebo: khaki; mannitol vs. placebo: medium purple; melatonin vs. placebo: lime green; methylprednisolone vs. placebo: magenta; midazolam vs. propofol: salmon; modafinil vs. placebo: gold; morphine vs. remifentanil: slate blue; n-acetylcysteine vs. placebo: tomato; placebo vs. pregabalin: dark turquoise.

**
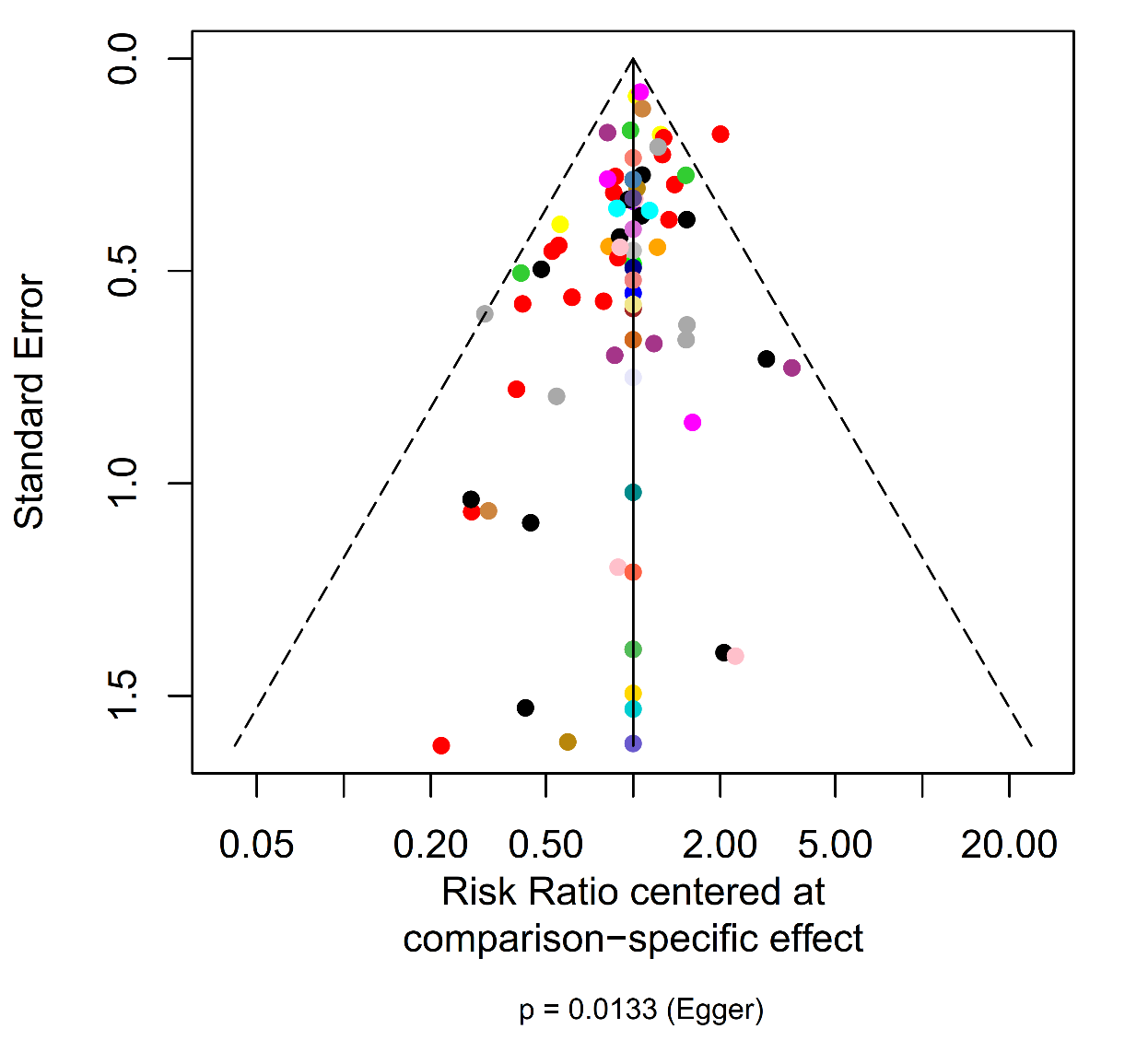
**
