# Supplementary material for: Effect of peri‐operative pharmacological interventions on postoperative delirium in patients having cardiac surgery: a systematic review and Bayesian network meta‐analysis
Source: Anaesthesia. 2025 Sep 1;81(2):274–87. doi: 10.1111/anae.16757 (PMC12803695; doi:10.1111/anae.16757)
Supplement: Supplementary file 5 — Table S1. Search strategy for each database. Table S2. Baseline characteristics of included studies. Table S3. Critical appraisal of individual studies. [file ANAE-81-274-s003.docx]

**Table S1**. Search strategy for each database.

| Cochrane | ("cardiac surgery" OR "cardiac surgeries" OR "cardiac valve surgery" OR "heart surgery" OR valve OR CABG OR "coronary artery bypass" OR "coronary artery bypass surgery" OR "open heart") AND ("Delirium" OR "postoperative delirium" OR "organic brain syndrome" OR "acute encephalopathy" OR "acute confusion") |
| --- | --- |
| Embase |  |
| PubMed |  |

**Table S2**. Baseline characteristics of included studies.

| **Author, Year** | **Country** | **Follow-up for Delirium** | **Intervention/Comparator Patients** | | **Mean age** | **Surgery** | **Intervention/ Comparator** | **Dosage of Intervention / Comparator** | **Route** | **Blinding** |
| --- | --- | --- | --- | --- | --- | --- | --- | --- | --- | --- |
| **Adabag et al (2008)** | USA | NA | 50/52 | | 71 | CABG (55%)  Valve (18.4%)  CABG and valve (14.5%)  Other (2%) | N-acetylcysteine  Placebo | I: 14 doses of liquid NAC (3 mL, 600 mg) | Oral | Double |
| **Al tmimi et al (2015)** | Belgium | 9 days | 21/21 | | 67.9 | Off-pump coronary artery bypass | Xenon  Sevoflurane | I: Xenon 50–60% in FI_O2_ =0.3–0.4  C: Sevoflurane 1.0–1.4% in oxygen and medical air (FI_O2_ = 0.3–0.4).  BIS 40-60 | Inhalation | Single |
| **Al tmimi et al (2017)** | Belgium | 5 days | 25/25 | | 67 | Off-pump coronary artery bypass | Xenon and propofol  Propofol | I: 30% xenon added to propofol TCI  C: General anesthesia with propofol TCI alone  BIS 40-60 | Inhalation | Single |
| **Al tmimi et al (2020)** | Belgium | 5 days | 96/94 | | 75.6 | On pump CPB | Xenon  Sevoflurane | I: 40-60% Xenon  C: 1.0-1.4% Sevoflurane  BIS 40-60 | Inhalation | Single |
| **Aykut et al (2024)** | Turkey | 1 day | 31/31 | | 61 | on-pump CABG | Sevoflurane  Propofol | I: 1.5-2% Sevoflurane  C: 50-100 μg kg/min/ propofol infusion  BIS 50-60 | Inhalation | Single |
| **Azeem et al (2018)** | Egypt | 7 days | 30/30 | | 66 | Coronary graft (65%)  Valve or valve-coronary graft (21.6%)  Redo surgery (8.3%)  Other (5%) | Dexmedetomidine  Morphine and midazolam | I: 1 μg/kg, followed by continuous infusion of 0.2–0.7 μg/kg/h  C: Morphine in a dose of 10–50 μg/kg/h with midazolam in a dose of 0.05 mg/kg up to 0.2 mg/kg repeated as needed | Intravenous | Double |
| **Balkanay et al (2015)** | Turkey | NA | 31 and 29 /28 | | 60.5 | CABG | Dexmedetomidine  Placebo | I: 4 and 8 μg/cc | Intravenous | Triple |
| **Baltali et al (2009)** | Turkey | 3 days | 29/29 | | 57.5 | CABG | Remifentanil  Morphine | I: Infusion 0.05 g/kg/min (1 mL/h), a bolus of 0.25 g/kg (1 mL), and a lockout time of 5 minutes  C: Infusion, 0.3 mg/h; bolus, 1 mg; and lockout time, 5 minutes | Intravenous | Double |
| **Chhabra et al (2024)** | India | NA | 50/50 | | 64.1 | CABG | Dexmedetomidine  Propofol | I: 1 mcg/kg over 10 minutes, followed by a maintenance infusion of 0.2-0.7 mcg/kg/hr  C: Continuous infusion at a rate of 2-5 mg/kg/hr | Intravenous | NA |
| **Chitnis et al (2022)** | Canada | 5 days | 34/33 | | 78.7 | CABG  AVR | Dexmedetomidine  Propofol | I: 0.5 mg/kg/h. This was titrated as required from 0 to 1.5 mg/kg/h  C: Infusion of 10% propofol at 25 to 50 mg/kg/min | Intravenous | Open label |
| **Corbett et al (2005)** | USA | NA | 43/46 | | 63 | CABG | Dexmedetomidine  Propofol | I: 1-ug/kg [actual body weight] loading dose over 15 mins, followed by a 0.4-ug/kg/hr/ infusion  C: or propofol (5-ug/kg/min intravenous infusion titrated within the range of 0.2–0.7 ug/kg/ hr or 5–75 ug·/kg/min | Intravenous | Single |
| **Coulson et al (2022)** | Australia | NA | 28/32 | | 68.8 | CABG (76.6%)  Valve (10%)  CABG and valve (13.3%) | Angiotensin-2  Noradrenaline | I: Starting at 2 ng/kg/min, with a titration range of 1–40 ng/kg/min to MAP of ≥ 70 mmHg  C: Starting at 0.02 μg/kg/min, with a titration range of 0.01–0.4 μg/kg/min to achieve the same MAP target | Intravenous | Double |
| **Dieleman et al (2012)** | Netherlands | NA | 2235/2247 | | 66.1 | CABG (39.5%)  CABG and valve (16.7%)  Single valve (25.2%)  Multiple valves (4%)  Others (14%) | Dexamethasone  Placebo | Single intraoperative dose of 1 mg/kg | Intravenous | Double |
| **Djaini et al (2016** | Canada | 5 days | 91/92 | | 72.5 | CABG (55.1%)  Valve (67.7%) | Dexmedetomidine  Propofol | I: 0.4 μg/kg bolus followed by 0.2 to 0.7 μg/ kg/h infusion  C: 25 to 50 μg/ kg/ min | Intravenous | Single |
| **Dong et al (2021)** | China | During hospitalization | 251/257 | | 64.7 | Without CPB (66.5%)  With CPB (33.4%) | Dexmedetomidine  Placebo | Up to 1.2 μg/kg/h until the RASS remained between − 1 and 0. | Intravenous | Double |
| **Duan et al (2023)** | China | 7 days | 144/145 | | 47.5 | AVR (18%)  MVR (45.3%)  AVR and MVR (37%) | Sevoflurane  Propofol | I: Sevoflurane 1.0–1.5 MAC  C: Propofol 2.0–3.0 mg/kg/h  BIS 40-60 and rSO_2_ of >80% of baseline value | Inhalation | Double |
| **Engoren et al (2001)** | USA | NA | 33/28/29 | | 63.4 | CABG (82.2%)  Valve (13.3%)  CABG and Valve (4.4%) | Fentanyl  Sufentanil  Remifentanil | Fentanyl 7–10 μg/kg for induction, with additional 1–2 μg/kg as needed  Sufentanil 1–4 μg/kg for induction, with additional 0.1–0.3 μg/kg as needed  Remifentanil infusion at 0.5–1.0μg/kg/min for induction, maintained at 0.05–1.0μg/kg/min, with boluses of 0.5–1.0 μg/kg as needed | Intravenous | NA |
| **Fang et al (2024)** | China | 5 days | 50/50 | | 67.5 | Off-pump CABG (35%)  Valve or aortic root surgery (65%) | Dexmedetomidine  Placebo | 0.3 μg/kg | Intranasal | Double |
| **Ford et al (2019)** | Australia | 7 days | 105/105 | | 68.3 | CABG or valve replacement | Melatonin  Placebo | 3 mg melatonin for 7 consecutive nights | Orally or via nasogastric tube | Double |
| **Gamberini et al (2009)** | Switzerland | 6 days | 57/56 | | 74.2 | CABG (52.2%)  Valve repair (27.8%) | Rivastigmine  Placebo | 3 doses of 1.5 mg of oral rivastigmine per day starting the evening before surgery and continuing until the evening of the sixth postoperative day | Oral | Double |
| **Hao et al (2024)** | China | 7 days | 89/90 | | 63 | Valve (42.4%)  CABG (13.4%)  Valve and CABG (10.6%) Great vessels (25.7%)  Others (7.2%) | Remifentanil  Dexmedetomidine | I: Initial dose of 0.05 μg/kg/min, titrated up in increments of 0.01 μg/kg/min to a maximum of 0.12 μg/kg/min  C: Initial dose of 0.5 μg/kg/h, titrated up in increments of 0.1 μg/kg/h to a maximum of 1.0 μg/kg/h. | Intravenous | Single |
| **Hemmati et al (2024)** | Iran | NA | 45/45 | | 64.1 | CABG | Mannitol serum and Ringer’s lactate  Ringer’s lactate | I: 200mL of 20% mannitol solution and 900mL of Ringer’s lactate solution  C: 1100mL of Ringer’s lactate solution | Intravenous | Double |
| **Hofland et al (2017)** | France, Germany, Italy, and The Netherlands | During hospital stay | 161/165/166 | | 64.3 | CABG | Xenon  Sevoflurane  Propofol | I: Maintenance with 40–65% xenon in the gas mixture, adjusted as needed. I: Maintenance with sevoflurane at an initial end-tidal concentration of 1.2%, adjusted to a maximum of 1.8%.  C: Propofol administered intravenously at an initial rate of 2–4 mg/kg/h, adjusted as needed.  BIS 40-60 | Inhalation  Intravenous | Single |
| **Huang et al (2024)** | China | 3 days | 36/35 | | 54 | Valve replacement surgery | Insulin  Normal saline interventions | I: 20 U of intranasal insulin (0.5 mL) twice daily, starting 2 days preoperatively  C: 0.5 mL of intranasal saline twice daily, starting 2 days preoperatively | Intranasal | Double |
| **Hudetz et al (2009)** | USA | Maximum of 5 days | 29/29 | | 64 | CABG (77.6%)  Valve (12%)  CABG and valve (10.4%) | Ketamine  Placebo | I:infusion of 0.5 mg/kg  C: Placebo in the same way | Intravenous | Double |
| **Huet et al (2024)** | France | 7 days | 165/166 | | 73 | CABG (54.7%)  AVR (42.3%)  MVR (11.5%)  CABG and valve (16%) | Dexmedetomidine  Placebo | 0.1–1.4 μg/kg/h, adjusted hourly to maintain a RASS of -1 to +1, from 8 PM to 8 AM, for up to 7 days or until ICU discharge. | Intravenous | Double |
| **Hulst et al (2019)** | Netherlands | During hospital stay | 129/132 | | 65 | CABG (35%)  Single non-CABG (39%)  Two or more procedures (26%) | Liraglutide  Placebo | 0.6 mg on the evening before surgery and 1.2 mg after induction of anaesthesia | Subcutaneous | Triple |
| **Javaherforoosh et al (2021)** | Iran | 2 days | 30/30 | | 61.5 | CABG | Melatonin  Placebo | 3 mg melatonin the evening before, the morning of surgery, and daily until postoperative day 2 | Oral | Double |
| **Javaherforoosh et al (2021)** | Iran | 7 days | 40/40 | | 59.7 | CABG | Melatonin and dexmedetomidine  Dexmedetomidine | I:3 mg melatonin daily for 5 days and dexmedetomidine 0.5 μg/kg bolus, then 0.3–0.5 μg/kg/h for 24 hours.  C: . 0.5 μg/kg bolus, then 0.3–0.5 μg/kg/h for 24 hours | Oral / Intravenous | Double |
| **Jiang et al (2023)** | China | 7 days | 341/343 | | 53.7 | Single valve (84.3%)  Multiple valves (8.9%)  Others (6.8%) | Sevoflurane or Desflurane  Propofol | I: 0.5–2 MAC during maintenance of anesthesia  C: 3–8 mg/kg/h throughout the procedure.  BIS 40-60 | Inhalation | Double |
| **Kamenshchikov et al (2022)** | Russia | NA | 48/48 | | 63.2 | Elective cardiac surgery with CPB | nitric oxide  Usual care | 40 ppm of gaseous nitric oxide via the CPB oxygenator during surgery | Inhalation | Double |
| **Kang et al (2018)** | China | NA | 50/47 | | 56.5 | MVR (49.5%)  AVR (25.7%)  MVR and AVR (24.7%) | Dexmedetomidine with isoflurane  Isoflurane | I:Dexmedetomidine 0.6 μg/kg bolus, then 0.2 μg/kg/h, plus isoflurane adjusted to BIS 40–50  C: Isoflurane adjusted to BIS 40–50. | Inhalation/Intravenous | Single |
| **Laaf et al (2021)** | Germany | During hospital stay | 10/10 | | 63.5 | Left ventricular assist device implantation | Selenium  Placebo | Selenium 300 mcg orally before surgery, followed by 3000 mcg IV after anesthesia induction, 1000 mcg IV at ICU admission, and 1000 mcg IV  daily in the ICU for up to 14 days | Oral / Intravenous | Double |
| **Later et al (2009)** | Netherlands | NA | 103/99/96 | | 65.1 | Heart surgery with the use of CPB. | Tranexamic acid or aprotinin  Placebo | Tranexamic acid group: 1 g loading dose, 500 mg added to CPB prime, and 400 mg/h continuous infusion during CPB  Aprotinin group: 2×10⁶ KIU loading dose, 2×10⁶ KIU added to CPB prime, and 5×10⁵ KIU/h continuous infusion during CPB | Intravenous | Double |
| **Li et al (2017)** | China | 5 days | 143/142 | | 66.9 | CABG (71.5%) Valve replacement (14.7%)  CABG and valve replacement (13.7%) | Dexmedetomidine  Placebo | 0.6 μg/kg bolus over 10 minutes, followed by 0.4 μg/kg/h during surgery and 0.1 μg/kg/h postoperatively until the end of mechanical ventilation | Intravenous | Double |
| **Likhvantsev et al (2020)** | Italy | 5 days | 84/85 | | 62.5 | CABG (56.2%)  Valve (34.3%)  CABG and valve (9.4%) | Dexmedetomidine  Placebo | I: 0.7 μg/kg/h during surgery (including CPB), reduced to 0.4 μg/kg/h in the ICU, with a range of 0.4–1.4 μg/kg/h at the physician's discretion | Intravenous | Double |
| **Liu et al (2016)** | China | During ICU stay | 29/32 | | 54.9 | Valve repair (9.8%)  Valve replacement (55.7%)  Valve repair and replacement (34.4%) | Dexmedetomidine  Propofol | I: Continuous infusion up to 1.5 μg/kg/h  C: Continuous infusion up to 50 μg/kg/min | Intravenous | Single |
| **Mahrose et al (2021)** | Egypt. | 5 days | 55/55 | | 66.6 | CABG | Dexmedetomidine  Dexmedetomidine plus Melatonin | I: Bolus of Dexmedetomidine 0.4 μg/kg over 20 minutes followed by 0.2–0.7 μg/kg/h infusion for maximum of 24 hours  C: Bolus of Dexmedetomidine 0.4 μg/kg over 20 minutes followed by 0.2–0.7 μg/kg/h infusion for maximum of 24 hours plus oral Melatonin tablet 5 mg the night before surgery and same dose was repeated every 24 hours for 3 postoperative days. | Oral / Intravenous | Single |
| **Maldonado et al (2009)** | USA | 3 days | | 30/60 | 58.2 | Cardiac Valve Surgery | Dexmedetomidine  Propofol  Midazolam | I: Dexmedetomidine loading dose: 0.4 g/kg, followed by a maintenance drip of 0.2 g/kg/hour– 0.7 g/kg/hour  II: propofol drip 25 g/kg/minute–50 g/kg/minute  III: midazolam drip 0.5mg/hour–2 mg/hour | Intravenous | Single |
| **Mansouri et al (2021)** | Iran | 3 Days | | 37/37 | 61.81 | CABG | Modafinil  Placebo | I: orally treated with doses of 200 mg of modafinil on the day of surgery, and on the morning of the day after surgery, the second dose of modafinil 200 mg  was given to patients  C: control group underwent a placebo with the same intervals. | Oral | Double |
| **Mardani et al (2013)** | Iran | 3 Days | | 43/50 | 62.5 | CABG (89.2%)  CABG + Valve (10.75%) | Dexamethasone  Placebo | I: 8 mg of Dexamethasone intravenous before induction of anesthesia followed by 8 mg every 8 h for 3 day  C: Placebo in the same way. | Intravenous | Double |
| **Missouri et al (2019)** | Iran | 3 Days | | 44/44 | 61.6 | CABG | Dexmedetomidine  Placebo | I: 1 μg/kg doses of dexmedetomidine immediately within 10 minutes and the infusion of 0.2-0.7 μg/kg/h of dexmedetomidine in a volume equivalent to 50cc by the syringe pump  C: Normal saline in same way | Intravenous | Double |
| **Momeni et al (2021)** | Denmark | 7 days | | 205/203 | 70.4 | Cardiac surgery with CPB | Propofol plus dexmedetomidine  Propofol plus placebo | I: propofol infusion at a dose rate of 1e3 mg kg1 h1 and dexmedetomidine infusion at a rate of 5 ml h1 corresponding to 0.4mgkg1 h1  C:propofol infusion at a dose of 1e3 mg kg1  h1 and placebo (saline 0.9%) at a rate of 5 ml h1 | Intravenous | Double |
| **Muellejans et al (2006)** | Germany | 3 days | | 39/33 | 65.75 | Bypass (67%)  Valve (23%) | Remifentanil plus propofol  Midazolam/fentanyl | I: remifentanil infusion was continued or started at an initial rate of 6 to 12 μg kg-1 h-1 and was increased depending on clinical need up to a maximum of 60μg kg-1 h-1 and propofol infusion starting at a rate of 0.5 to 1.0 mg kg-1 h-1.  C:bolus dose of fentanyl of 1 to 2 μg kg-1, followed by an infusion at an initial rate of 1 to 2 μg kg-1 h-1 and midazolam range from 0.03 to 0.2 mg kg-1, patients commonly received a 2 mg bolus at the lower end of this range. This was followed by an infusion at an initial rate of 0.02 to 0.04 mg kg-1 h-1. | intravenous | Double |
| **Park et al (2014)** | South Korea | 3 days | | 67/75 | 52.7 | CABG | Dexmedetomidine  Remifentanil | I: dexmedetomidine (loading dose, 0.5 μg/kg; maintenance dose 0.2 to 0.8 μg/kg/hr)  C: remifentanil (range, 1,000 to 2,500μg/hr). | Intravenous | Double |
| **Pesonen et al (2011)** | Finland | 5 days | | 35/35 | 79.55 | CABG plus CPB (65.7%)  Valve (30%)  Others (4.3%) | Pregabalin  Placebo | I: Patients were premedicated orally 1 h before surgery with lorazepam (0.02–0.03 mg kg21) and the study drug, pregabalin 150 mg (Lyricaw 75 mg capsule, Pfizer GmbH, Freiburg, German) Beginning on the first postoperative morning, patients received 75 mg pregabalin  C: placebo twice daily until the fifth postoperative day. | Intravenous | Double |
| **Prakanrattana et al (2007)** | Thailand | N/A | | 63/63 | 61 | CABG (70%)  Valve (28%)  Others (2%) | Risperidone  Placebo | I: Risperidone 1mg orally  C: Placebo sublingually | Oral | Double |
| **Preveden et al (2023)** | Serbia | 5 Days | | 60/60 | 64.9 | Cardiac Surgery using CPB | Dexmedetomidine  Propofol | I: dexmedetomidine infusion in doses 0.2-0.7mcg/kg/h. Dexmedetomidine infusion was discontinued before weaning from MV and extubation. For patients requiring MV longer than 24 hours, dexmedetomidine infusion was substituted with propofol.  C: propofol infusion in doses 1-2 mg/kg/h. Propofol infusion was also discontinued before weaning from MV and extubation. | Intravenous | Double |
| **Priye et al (2015)** | India | 1 day | | 32/32 | 43.25 | Elective CABG | Placebo  Dexmedetomidine | I: received a 12 h infusion of normal saline  C: received a 12 h infusion of dexmedetomidine  0.4 μg/kg/h without a loading dose. | Intravenous | Double |
| **Qu et al (2023)** | USA | 3 days | | 188/206 | 68.75 | CABG (19%)  Valve + CABG (10%)  Valve (50%)  Others (21%) | Dexmedetomidine  Placebo | I: dexmedetomidine (1μg/kg over 40 min, maximal dose of 80 μg  C: Placebo same way | Intravenous | Double |
| **Racman et al (2023)** | Slovenia | 3 days | | 37/34 | 83.25 | TARV | Propofol  Dexmedetomidine | I: 0.5-2.5mg/kg/h of propofol by continuous intravenous infusion with an infusion pump  C: Dexmedetomidine group first received a loading dose of 0.5 mg/kg of dexmedetomidine over 10 minutes, followed by continuous intravenous infusion of 0.2-1.0 mg/kg/h of dexmedetomidine with an infusion pump | Intravenous | Double |
| **Rohm et al (2008)** | Germany | 10 days | | 35/35 | 65.5 | CABG (90%)  Valve (10%) | Sevoflurane  Propofol | I: sevoflurane, a bolus of 1–2 mL was given and an infusion rate of 2–6 mL/h adjusted to obtain end tidal concentrations of 0.5–1 vol%.  C:intravenous propofol 2% was given initially in a dosage of 2 mg/kg/h with further adjustments to a maximum of 4 mg/kg/h. | Intravenous | Single |
| **Roque et al (2021)** | Canada | 1 day | | 37/39/39 | 67.61 | CABG (60.8%)  Valve (12.1%)  Others (27.1%) | Placebo  Insulin 40 Ui  Insulin 80 Ui | I: Intranasal normal saline  C: 40 IU intranasal insulin  C:80 IU intranasal insulin. | Intranasal | Single |
| **Royse et al (2011)** | Australia | 90 days | | 89/91 | 62.8 | CABG | Desflurane  Propofol | Propofol concentration infusion from 1.5 to 3 lg.ml | Inhalation  Intravenous | Single |
| **Royse et al (2017)** | Australia | 3 days | | 236/246 | 73.9 | Valve (49%)  CABG on pump (24%)  CABG plus Valve (19%)  Aortic surgery (20%) | methylprednisolone  Placebo | I: 250mg methylprednisolone at induction and 250mg methylprednisolone before cardiopulmonary bypass.  C: Placebo in the same way | Intravenous | Double |
| **Rubino et al (2010)** | Italy | ICU length of stay | | 15/15 | 62.6 | acute type-A aortic  dissection | Clonidine  Placebo | I: clonidine (0.5 µ/kg/h bolus, followed by continuous infusion at 1–2 µ/kg/h)  C: Placebo in tha same way | Intravenous | Double |
| **Sauer et al (2014)** | Netherlands | 4 days | | 367/370 | 66.5 | CABG (36%)  Valve (60%)  Others (4%) | Dexamethasone  Placebo | I: 1 mg/kg IV injection of dexamethasone (maximum 100 mg)  C: Placebo in the same way | Intravenous | Double |
| **Shehabi et al (2009)** | Australia | 5 days | | 152/147 | 71.3 | CABG (60%)  Valve plus CABG (30%)  Others(10%) | Dexmedetomidine  Morphine | I: Dexmedetomidine 0.1–0.7 µg · kg1 ·ml1  C: Morphine 10–70 µg · kg1 · ml1 | Intravenous | Double |
| **Sheikh et al (2018)** | India | ICU length of stay | | 30/30 | 34.5 | Elective cardiac surgery. | Dexmedetomidine  Propofol | I: Dexmedetomidine (1 μg/kg diluted in 100 ml of normal  saline over 10 min), followed by infusion (0.2–0.6 μg/kg/h).  C: Propofol infusion at the rate of 0.25–1 mg/kg/h. | Intravenous | Double |
| **Shi et al (2021)** | China | 7 days | | 148/149 | 71.5 | PCI | Melatonin  Placebo | I: 3 mg/day Melatonin within 7 days after PCI.  C: Placebo in the same way | Oral | Double |
| **Shokri et al (2020)** | Egypt | 14 days | | 144/142 | 64 | CABG | Dexmedetomidine  Clonidine | I: Dexmedetomidine continuous infusion of 0.7–1.2 μg/kg/h; then, adequacy of sedation and analgesia were evaluated after 45–60 min. If the RASS score ranged from +1 to +4, the infusion rate of dexmedetomidine was increased by 0.1–0.2 μg/kg/h every 30 min up to the maximum dose of 1–1.4 μg/kg/h based on the patient's actual body weight.  C:0.5 μg/kg intravenously (IV) slowly, over a period of 10–15 min, followed by a continuous IV infusion of 1–2 μg/kg/h if the RASS ranged from +1 to +4, which was continued until tracheal extubation. It was prepared as follows: Clonidine (Catapres ampoules 150μg/ml, Boehringer Ingelheim Ltd, Berkshire, UK) was diluted in 0.9% saline and drawn up in 50-ml syringe to a concentration of 15 μg/ml. | Intravenous | Double |
| **Shu et al (2017)** | China | N/A | | 30/30 | 47.3 | Valve Replacement | Dexmedetomidine  Placebo | I: Dexmedetomidine group were intravenously injected with Dexmedetomidine at 1.0 μg/kg in 10 min, followed with continuous transfusion of 0.5 μg/kg/h until the end of surgery.  C:Placebo in the same way | Intravenous | Double |
| **Siripoonwothai et al (2021)** | Thailand | 1 day | | 32/32 | N/A | CPB | Ketamine  Propofol | I: infusion of ketamine at 1 mg/kg/h,[13] fentanyl at 0.5‐1mcg/kg/h and cisatracurium at 1.5 mcg/kg/min during CPB.  C: infusion of propofol at 1.5‐6 mg/kg/h,[14] fentanyl at 0.5‐1 mcg/kg/h and cisatracurium at 1.5 mcg/kg/min. | Intravenous | Double |
| **Soh et al (2020)** | South Korea | 2 day | | 54/54 | 65 | Ascending aorta and aortic arch replacement (61%)  Descending thoracic aorta replacement (15%)  Others (24%) | Dexmedetomidine  Placebo | I: Dexmedetomidine 200 mg was mixed with 0.9% saline to achieve a concentration of 4 mg/ml1, which was continuously infused at a rate of 0.4 mg kg1 h1 for 24 h starting immediately after anaesthetic induction.  C: Placebo in the same way | Intravenous | Double |
| **Stoppe et al (2013)** | Germany | 1 day | | 15/15 | 67 | CABG | Xenon  Sevoflurane | I: xenon (45–50 vol%)  C: sevoflurane (1–1.4 vol%). | Inhalation | Single |
| **Stoppe et al (2023)** | Canada and Germany | ICU length of stay | | 697/697 | 68.25 | CABG plus valve/multivalve (58%)  CABG (4%)  Multivalve (12%)  Others (26%) | selenium  Placebo | I: either received 2000μg/L of intravenous selenium (sodium selenite; Selenase) within 30 minutes after induction of anesthesia and prior to initiation of CPB, then 2000μg/L of intravenous selenium immediately on admission to the postoperative ICU, then 1000 μg/L of intravenous selenium each successive morning while in ICU  C: placebo at the same time points for a maximum of 10 days | Intravenous | Double |
| **Stupica et al (2024)** | Slovenia | ICU length of stay | | 20/20 | 68.7 | CABG | Methylprednisolone | I: 1g of methylprednisolone added in CPB priming solution  C:Usual care; without methylprednisolone during CPB | Intravenous | Single |
| **Subramaniam et al (2019)** | USA | ICU length of stay | | 29/30/31/30 | 69 | CABG (65%)  CABG plus Valve (35%) | Propofol plus acetaminophen  Propofol  Dexmedetomidine Plus acetaminophen  Dexmedetomidine | I: Dexmedetomidine group received an IV bolus dose of 0.5 to 1 μg/kg during chest closure, followed by a maintenance infusion of 0.1 to 1.4 μg/kg per hour.  C: Propofol group received a maintenance dose of 20 to 100 μg/kg per minute. | Intravenous | Double |
| **Susheela et al (2017)** | USA | 3 days | | 3/3/3/3 | N/A | CABG | Propofol plus acetaminophen  Propofol  Dexmedetomidine Plus acetaminophen  Dexmedetomidine | I: Propofol infusion was titrated to 25–100 μg/kg/min.  C: Dexmedetomidine infusion was given after chest closure at a dose of 0.1–1.0 μg/kg/hr. s | Intravenous | Double |
| **Turan et al (2020)** | USA | 5 days | | 398/396 | 62.5 | Valve (54%%)  Valve plus CABG (46%) | Dexmedetomidine  Placebo | I: Dex of 0·1 μg/kg per h; at the end of bypass,  the dose was increased to 0·2 μg/kg per h. Postoperative to 0·4 μg/kg per h which was maintained until 24 h after the infusion began.  C: Placebo same way | Intravenous | Double |
| **Varsha et al (2024)** | India | 7 days | | 35/34 | 59.4 | CABG plus CPB | Propofol  Sevoflurane | N/A | Intravenous/Inhalation | Single |
| **Wang et al (2023)** | China | 7 Days | | 326/326 | 53.9 | Isolate Valve (57%)  Multiple Valve (43%) | Dexmedetomidine  Placebo | I: 0.6 lg kg1 dexmedetomidine over 10 min and then a continuous infusion of dexmedetomidine 0.4 lg.kg1.h1 until the end of surgery.  C: Placebo in the same way | Intravenous | Double |
| **Whitlock et al (2015)** | Canada | N/A | | 3755/3752 | 67.4 | Isolate Valve (65%)  Others (35%) | Methylprednisolone  Placebo | I: Methylprednisolone dose was 30 mg/kg (>2 g for a 70 kg patient). We decided, however, to use a cumulative dose of 500 mg of methylprednisolone given intraoperatively  C: Placebo in the same way | Intravenous | Double |
| **Wittwer et al (2023)** | Australia | N/A | | 25/24 | 76.2 | Complex cardiac surgery involving multiple valves or redo sternotomy | Ketamine  Propofol | I: Ketamine group received 1–2 mg/kg of intravenous  ketamine for induction of anesthesia  C: propofol group received 0.5–1 mg/kg of intravenous propofol | Intravenous | Single |
| **Xiong et al (2024)** | China | 7 days | | 56/56 | 52 | Isolate Valve (90%)  Multiple Valve (10%) | Esketamine  Placebo | I: Solution containing 0.25 mg/kg  esketamine with a total volume of 10 mL.  C: Placebo in the same way | Intravenous | Triple |

**Table S3**. Critical appraisal of individual studies according to the Cochrane Collaboration’s tool for assessing risk of bias (RoB-2) in randomized trials.

| **Study** | **Domain 1** | **Domain 2** | **Domain 3** | **Domain 4** | **Domian 5** | **Overall** |
| --- | --- | --- | --- | --- | --- | --- |
| Adabag 2008 | Low | Low | Low | Low | Low | Low |
| Al Tmimi 2015 | Low | Low | Low | Low | Low | Low |
| Al Tmimi 2017 | Low | Low | Low | Low | Low | Low |
| Al Tmimi 2020 | Low | Low | Low | Low | Low | Low |
| Aykut 2024 | Some concerns | Low | Low | Low | Low | Some concerns |
| Azeem 2018 | Low | Low | Low | Low | Low | Low |
| Balkanay 2015 | Some concerns | Low | Low | High | Low | High |
| Baltali 2009 | Low | Low | Low | Low | Low | Low |
| Chhabra 2024 | Some concerns | Low | Low | Some concerns | Low | Some concerns |
| Chitnis 2022 | Low | Low | Low | Low | Low | Low |
| Corbett 2005 | Some concerns | Some concerns | Some concerns | Some concerns | Some concerns | High |
| Coulson 2022 | Some concerns | Low | Low | Some concerns | Some concerns | Some concerns |
| Dieleman 2012 | Low | Low | Low | Some concerns | Low | Some concerns |
| Dijaini 2016 | Low | Low | Low | Low | Low | Low |
| Dong 2021 | Low | Low | Low | Low | Low | Low |
| Duan 2023 | Low | Low | Low | Low | Low | Low |
| Engoren 2001 | Some concerns | Low | Low | Some concerns | Some concerns | Some concerns |
| Fang 2024 | Some concerns | Low | Low | Low | Some concerns | Some concerns |
| Ford 2019 | Some concerns | Low | Low | Low | Low | Some concerns |
| Gamberini 2009 | Low | Low | Low | Some concerns | Some concerns | Some concerns |
| Gao 2021 | Some concerns | Low | Low | Low | Low | Some concerns |
| Hao 2024 | Low | Low | Low | Low | Low | Low |
| Hofland 2017 | Low | Low | Low | Low | Low | Low |
| Huang 2024 | Some concerns | Some concerns | Low | Low | Low | Some concerns |
| Hudetz 2009 | Some concerns | Low | Low | Low | Low | Some concerns |
| Huet 2024 | Low | Low | Low | Low | Low | Low |
| Hulst 2019 | Low | Low | Low | Low | Low | Low |
| Jiang 2023 | Low | Low | Low | Low | Low | Low |
| Kamenshchikov 2022 | Low | Low | Low | Low | Low | Low |
| Kang 2018 | Low | Some concerns | Low | Low | Low | Some concerns |
| Kim 2021 | Low | Low | Low | Low | Low | Low |
| Laaf 2021 | Low | Low | Low | Low | Low | Low |
| Later 2009 | Low | High | Low | Some concerns | Low | High |
| Li 2017 | Low | Low | Low | Low | Low | Low |
| Likhvantsev 2020 | Low | Low | Low | Low | Low | Low |
| Liu 2016 | Low | Low | Low | Low | Low | Low |
| Mahrose 2021 | Some concerns | Low | Low | Low | Low | Some concerns |
| Maldonado 2009 | Some concerns | Low | Low | High | Low | High |
| Mansouri 2021 | Some concerns | Some concerns | Low | High | Low | High |
| Mardani 2013 | Low | Some concerns | Low | Low | Low | Some concerns |
| Maslakpak 2024 | Low | Some concerns | Some concerns | Low | Low | Some concerns |
| Massoumi 2019 | Low | Low | Low | Low | Low | Low |
| Missouri 2019 | Low | Low | Low | Low | Low | Low |
| Momeni 2021 | Low | Low | Low | Low | Low | Low |
| Muellejans 2006 | Some concerns | Some concerns | Low | High | Low | High |
| Park 2014 | Some concerns | Low | Low | Low | Low | Some concerns |
| Pesonen 2011 | Low | Low | Low | Low | Low | Low |
| Prakanrattana 2007 | Low | Low | Low | Low | Low | Low |
| Preveden 2023 | Some concerns | Low | Low | Low | Low | Some concerns |
| Priye 2015 | Low | Low | Low | Low | Low | Low |
| Qu 2022 | Low | Low | Low | Low | Low | Low |
| Racman 2023 | Some concerns | Low | Low | Low | Low | Some concerns |
| Rohm 2008 | Some concerns | Low | Low | High | Low | High |
| Roque 2021 | Some concerns | Low | Low | Low | Low | Some concerns |
| Royse 2011 | Low | Low | Low | Low | Low | Low |
| Royse 2017 | Low | Low | Low | Low | Low | Low |
| Rubino 2010 | Some concerns | Some concerns | Low | High | Low | High |
| Sauer 2014 | Low | Low | Low | Low | Low | Some concerns |
| Shehabi 2009 | Low | Low | Low | Low | Low | Low |
| Sheikh 2018 | Low | Low | Low | High | Low | High |
| Shi 2021 | Low | Low | Low | Low | Low | Low |
| Shokri 2020 | Low | Low | Low | Low | Low | Low |
| Shu 2017 | Some concerns | Some concerns | Low | Low | Low | Some concerns |
| Siripoonwothai 2021 | Low | Low | Low | Low | Low | Low |
| Soh 2022 | Some concerns | Some concerns | Low | Some concerns | Low | Some concerns |
| Stoppe 2013 | Some concerns | Low | Low | Low | Low | Some concerns |
| Stoppe 2023 | Some concerns | Low | Low | Low | Low | Some concerns |
| Stupica 2024 | Low | Low | Low | Some concerns | Low | Some concerns |
| Subramaniam 2019 | Some concerns | Low | Low | Low | Low | Some concerns |
| Susheela 2017 | Some concerns | Low | Low | Low | Low | Some concerns |
| Turan 2020 | Low | Low | Low | Low | Low | Low |
| Varsha 2024 | Low | Low | Low | Low | Low | Low |
| Wang 2023 | Low | Low | Low | Low | Low | Low |
| Whitlock 2015 | Low | Low | Low | Low | Low | Low |
| Wittwer 2023 | Low | Low | Low | Low | Low | Low |
| Xiong 2024 | Low | Low | Low | Low | Low | Low |
| Zadeh 2021 | Some concerns | Low | Low | Low | Low | Some concerns |
| Zadeh 2023 | Low | Low | Low | Low | Low | Low |
